# Gut microbiota regulates the brain metabolism of sexually mature drones

**DOI:** 10.1128/spectrum.02536-24

**Published:** 2025-06-05

**Authors:** Jun Zhang, Ya-Zhou Zhao, Meng-Shang Hou, Qi-He Tang, Yan-Tao Pang, Xi-Jie Li, Jian Xiong, Chong-Hui Zhao, Qi Huang, Shao-Jiang Tian, Zhi-Xiang Dong, Zhen-Xing Liu, Jun Guo

**Affiliations:** 1 Faculty of Life Science and Technology, Kunming University of Science and Technology, Kunming, China; 2 State Key Laboratory of Resource Insects, Institute of Apicultural Research, Chinese Academy of Agricultural Sciences, Beijing, China; 3 Sericulture and Apiculture Research Institute, Yunnan Academy of Agricultural Sciences, Mengzi, China; 4 Yunnan Zhongfeng Technology Development Co. Ltd., Kunming, Yunnan, China; 5 Kunming Maternity and Child Care Hospital, Kunming, Yunnan, China; 6 Zhejiang Jiangshan Healthy Apiculture Co., Ltd., Jiangshan, Zhejiang, China; China Agricultural University, Beijing, China

**Keywords:** drone, gut microbiota, sexual maturation, brain, metabolomics

## Abstract

Previous studies have found that gut microbiota and metabolites may be crucial in insect sexual maturation. For example, during sexual maturity, the increased dopamine in the drone’s brain can affect its reproductive behavior, such as flight, mating, and so on. In addition, gut microbiota can affect brain functions such as learning and memory through metabolites. However, it is currently unclear about the changes in gut microbiota during drone sexual maturation and whether they can affect brain function through their metabolites. Thus, we analyzed gut microbiota and brain metabolome in drones during sexual maturation based on multi-omics and identified markers associated with sexual maturation. In addition, we also explored the potential correlation between differentially expressed microorganisms and metabolites before and after sexual maturation. The results showed significant changes in microbes (*Lactobacillus*, *Chaetothyriales*, *Mortierella*, and *Entrophospora*), and metabolite pathways (glycerophospholipids), with substantial changes. *Lactobacillus* and *Chaetothyriales* showed the most significant changes and therefore can be used as marker bacteria in the sexual maturation of drones. In addition, PS (16:0/20:1(11Z)), PE (P-18:0/20:4), and LysoPC (P-18:0/0:0) showed significant differences in the variation of substances, which can be used as markers related to memory in sexually mature drones. This suggests that the intestinal flora of sexually mature drones undergoes substantial changes and has a stronger glycerophospholipid metabolism. Furthermore, microbial-metabolite correlation analysis revealed that *Lactobacillus*, *Aspergillus*, *Mortierella*, and *Pleosporales* were strongly correlated with PC and PE in the glycerophospholipid metabolic pathway. Therefore, we hypothesize that these four species may affect drones' brain function by influencing glycerophospholipid metabolism, thereby affecting their learning and memory behavior. These findings provide new insights into the interactions between sexual maturation, gut microbes, and brain metabolism in drones.

**IMPORTANCE:**

Learning and memory are essential for drones to mate with the queen, for example, drones need to learn and memorize the sex pheromone of the queen through olfaction to find the queen for mating. However, research in this area is still limited. We investigated the factors affecting learning and memory behaviors in sexually mature drones by analyzing the gut microbiota and brain metabolites of drones of different ages. In this study, we explored the diversity of gut microbiota and changes in brain metabolism during sexual maturation in drones. Through a comprehensive analysis of gut microbiota and brain metabolism in drones, we found that gut microbiota may influence learning and memory in drones by regulating the content of glycerophospholipids in the brain. These findings provide valuable insights into the use of gut microbiota to regulate learning and memory in sexually mature drones and thereby enhance their mating rate with queens.

## INTRODUCTION

Drones play an important role in honeybee reproduction because their main role is to mate with unmated queens (1). Learning and memory are essential for drones to mate with queens, for example, drones need to learn and memorize the sex pheromones of queens through olfaction to find the queens for mating (2). However, research in this area is still limited. It has been gradually recognized that gut microbes can influence host brain function through their metabolites. For example, Zheng et al. (3) demonstrated that honeybee gut *Lactobacillus* was able to activate host aryl hydrocarbon receptors through the conversion of tryptophan to indole derivatives, thereby promoting memory behavior. Li et al. (4) also demonstrated that *Lactobacillus* Firm-5 was able to increase glycerophospholipids in the bumblebee brain to improve its long-term memory. Research on the process of sexual maturation in drones is currently focused on the reproductive system, including the expression of different genes in the reproductive organs during sexual maturation (5). Changes in testicular organization and structure (6) and the effects of environmental factors and pesticides on drone spermatozoa (7, 8). In addition, it has been shown that dopamine increases in the brains of drones during sexual maturation, which in turn affects the flight and mating behavior of drones. However, the intestinal flora and related brain metabolism during sexual maturation have not been reported, and it is not known whether the intestinal flora during sexual maturation regulates the function of the brain of drones through metabolites.

In this study, we collected intestinal and brain samples from three groups of drones of varying ages (3, 8, and 20 days old) during sexual maturation. Subsequently, we employed 16S rRNA and ITS techniques to identify gut bacteria and fungi, and we analyzed the non-targeted metabolomic profiles of drone brains. Utilizing these methods, we observed significant changes in *Lactobacillus* and glycerophospholipid metabolism, as well as a notable correlation between the two. Based on the relevant literature and our current findings, we hypothesize that the substantial increase in *Lactobacillus* during sexual maturation facilitates the synthesis of additional glycerophospholipids, thereby increasing the glycerophospholipid content in the brains of drones and influencing the memory of sexually mature drones. These research findings help us further understand the changes in gut microbiota and brain metabolites of drones during sexual maturation, and use these results to regulate drone reproductive behavior.

## MATERIALS AND METHODS

### Collection of drones

This experiment was conducted in June 2023 at the apiary of Kunming University of Technology. Six healthy, disease-free honey bee colonies (*Apis mellifera ligustica*) were selected. Their colony strength, capper area, and food supply were consistent among the selected colonies. The evening before the experiment commenced, a drone brood frame was introduced into each colony that had begun laying drone eggs and cleaned for 12 h. After that, we used a queen oviposition controller to induce the queen to lay eggs on the empty drone frame, and after 48 h (9), the queen bee was removed, and the frame was placed in the upper box (queen-free) for further incubation and development (10). The frames containing drone pupae were removed from the hives 1 day before the drones emerged from the hive, and placed in a constant temperature and humidity box (with a temperature of 35°C, relative humidity of 95%, and no light). Newly fledged drones (0 days old) were painted on their thorax to determine their age, and then the tagged 432 drones were then returned to the original colony, placing 72 drones in each colony and adding a relay box to the hive to facilitate flight and defecation by the drones. To avoid worker bees driving drones and drones out of the hive, pollen traps were installed on the hive door of the colony, and the colony was rewarded with feeding (10). Drones usually recognize the nest and fly on the eighth day after emergence (11), at this time they are not yet sexually mature. We chose to sample on days 3 and 8 as the sample before sexual maturity. The effective mating age of drones is usually between days 12 and 35 after emergence (12), so we collected samples at day 20 as the sample after sexual maturity. Six labeled drones were also randomly collected from each hive, quickly frozen in liquid nitrogen, and stored at −80°C for subsequent experiments.

### Dissection of the drone intestine and brain

All experiments were conducted in a sterile ultra-clean bench environment. Initially, the drones were retrieved from the −80°C refrigerator and placed on ice for thawing. Subsequently, the drone samples underwent a 1-min immersion in 75% alcohol to eliminate surface bacteria after thawing, followed by a 30-s immersion in double-distilled water. The drones were then embedded in wax disks. Dissection of the drones commenced with scissors from the caudal end along the abdominal sides. Subsequently, the entire intestinal tissue was extracted, transferred to a 1.5 mL sterile centrifuge tube, three intestines as one replicate, a total of six replicates, rapidly frozen with liquid nitrogen, and stored at −80°C for preservation (13) for 16 s and ITS sequencing, also labeled X3, X8, X20, and F3, F8, F20, respectively. To compare the changes in brain metabolism of drones before and after sexual maturity, we chose days 8 and 20 as comparative objects. The frozen brains were dissected on ice using a dissecting microscope to excise the head cuticle and delicately extract the hypopharyngeal glands, salivary glands, three single eyes, and two compound eyes. The isolated brains were placed in 1.5 mL sterile centrifuge tubes, three brains for one replicate, for a total of six replicates, rapidly frozen in liquid nitrogen, preserved at −80°C for storage, and labeled N8 and T20.

### Gut microbiota DNA extraction and 16s and ITS sequencing

The intestines of the drones were transferred to another 1.5 mL sterile centrifuge tube containing 100 µL of double-distilled water and 0.1 mM ceramic beads for DNA extraction. Gut samples from drones were homogenized in a tissue lyser and then processed for DNA extraction using the Insect DNA Kit (Do926-02; Omega, Inc., USA). Total DNA was eluted in 50 µL of elution buffer according to the manufacturer’s instructions, and the extracted DNA was subjected to quality evaluation by NanoDrop2000 (Thermo Science, Wilmington, USA) and 2% agarose gel electrophoresis to measure and evaluate the concentration and quality of the extracted DNA.

The bacterial universal primers 338F (5′-ACTCCTACGGGGAGGCAGCAG-3′) and 806R (5′-GGAC-TACHVGGGTWTCTAAT-3′) (14) were employed for the bacterial PCR amplification targeting the highly variable region V3–V4 of the 16S rRNA gene. For the study of Fungi communities, sequencing was carried out targeting the second internal transcribed spacer (ITS2 region). In this case, amplification was performed using the primers ITS3: 5′-GCATCGATGAAGAACGCAGC-3′ and ITS4: 5′-TCCTCCGCTTA TTGATATGC-3′ (15, 16). The amplification process included denaturation at 95°C for 3 min, followed by 27 cycles of denaturing at 95°C for 30 s, annealing at 55°C for 30 s, and extension at 72°C for 45 s, single extension at 72°C for 10 min, and termination at 4°C. Subsequently, the PCR products were purified by electrophoresis using 1 × TAE agarose gel at 2% concentration, and the target bands were recovered by shearing, followed by recovery using the Thermo Scientific GeneJET Gel Recovery Kit.

The library was constructed using Thermofisher’s Ion Plus Fragment Library Kit 48 rxns, and after the constructed library was qualified by Qubit quantification and library testing, it was sequenced using Thermofisher’s Ion S5TMXL. The raw fastq data obtained from sequencing were first processed Trimmomatic (http://www.usadellab.org/cms/index.php? page = trimmomatic) for data quality control and then spliced the paired-end data using Flash (https://ccb.jhu.edu/software/FLASH/index.shtml). The splicing criteria were as follows: (i) average quality score <20/50 bp reads were truncated; (ii) the paired-end sequences were spliced based on the principle that the overlap length was greater than 10 bp and the base mismatch rate was ≤20%; and (iii) sequence isolation was conducted for each sample according to the barcodes (exact match) and primers (two nucleotide mismatches were allowed), with removal of sequences containing ambiguous bases. Subsequently, operational taxonomic units (OTUs) with 97% sequence similarity were clustered using UPARSE software (http://www.drive5.com/uparse/). The OTUs were categorized and analyzed by the Ribosomal Database Project (RDP, version 2.11) Classifier algorithm (https://sourceforge.net/projects/rdp-classifier/), and a 70% confidence threshold was used to compare the OTUs with the SILVA 128 16S rRNA database, and Qiime software (https://qiime.org/install/index.html) was used to calculate the alpha diversity and beta diversity (17).

### Preparation of metabolome samples

A 50 mg sample of drone brains was carefully weighed into a 2 mL centrifuge tube, and 6-mm-diameter grinding beads were meticulously included. Extracts (400 µL; methanol:acetonitrile = 1:1, vol/vol) of four internal standards (L-2-chlorophenylalanine) were added. The extracts underwent grinding with a cryogenic tissue grinder at −10°C and 50 Hz for 6 min, followed by sonication extraction at 5°C and 40 kHz for 30 min. After being stored at −20°C for 30 min, the samples underwent centrifugation at 13,000 × *g* and 4°C for 15 min, followed by transfer of the supernatant to a syringe vial equipped with an internal cannula for subsequent analysis. Furthermore, 20 µL of supernatant was extracted from each sample, combined, and utilized as a quality control sample as described in reference (13).

### Untargeted metabolomics profiling of the drone brain

The instrumental platform for this LC-MS analysis was an ultra-high performance liquid chromatography-tandem Fourier transform mass spectrometer (UHPLC-Q Exactive HF-X System; Thermo Science). The chromatographic column was ACQUITY UPLC HSS T3 (100 mm × 2.1 mm i.d., 1.8 µm; Waters Corporation, Milford, USA), the mobile phase A was 95% water + 5% acetonitrile (containing 0.1% formic acid), and the mobile phase B was 47.5% acetonitrile + 47.5% isopropanol + 5% water (containing 0.1% formic acid). The injection volume was 3 µL, and the column temperature was 40°C. The sample was ionized by electrospray ionization.

The sample was subjected to electrospray ionization, and the mass spectra were obtained by positive and negative ion scans. The scanning range was 70–1,050 *m/z*, the sheath gas flow rate was 50 arb; the auxiliary gas flow rate was 13 arb; the heating temperature was 425°C; the capillary temperature was 325°C; the spray voltage was (+3,500 and −3,500 V); and the lens voltage was 50 V. We first injected three quality control samples to equilibrate the system and column. During the analysis, one QC sample was injected after every three samples to monitor the stability of the instrument.

### Analysis of untargeted metabolomics data

After onboarding, the LC-MS raw data were imported into the metabolomics processing software Progenesis QI (Waters Corporation, Milford, USA) for baseline filtering, peak identification, integration, retention time correction, peak alignment, ultimately resulting in a data matrix of retention times, mass-to-charge ratios, and peak intensities. Concurrently, the MS and MS/MS information was combined with the metabolic information. The MS and MS/MS mass spectrometry information was matched with the metabolic public databases, HMDB (http://www.hmdb.ca/) and Metlin (https://metlin.scripps.edu/), as well as majorbio’s libraries, to obtain metabolite information.

The data matrix obtained after searching the library was uploaded to the majorbio’s cloud platform (http://cloud.majorbio.com) for analysis. The data matrix was first preprocessed as follows: the 80% rule was used to remove missing values, that is, variables with more than 80% of non-zero values in at least one set of samples were retained, and the missing values were then filled with the smallest values in the original matrix. To minimize errors due to sample preparation and instrumental instability, the response intensities of the sample peaks in the mass spectrometry were normalized using the sum-normalization method, resulting in normalized data matrices. Additionally, variables with a relative standard deviation >30% were removed, and log10 transformation was performed to obtain the final data matrix for subsequent analysis.

Next, principal component analysis (PCA) and orthogonal partial least squares discriminant analysis (OPLS-DA) were performed on the preprocessed data matrices using the ropls package in R (version 1.6.2), and the stability of the model was assessed using seven cycles of cross-validation. The selection of significantly different metabolites was determined based on the variable importance in projection (VIP) values obtained from the OPLS-DA model and the Student’s *t* test *P* value; metabolites with VIP >1, *P* < 0.05 were considered significantly different metabolites.

Differential metabolites were obtained by metabolic pathway annotation via the KEGG database (https://www.kegg.jp/kegg/pathway.html) to obtain the pathways in which the differential metabolites are involved. The Python package scipy.stats was used to perform pathway enrichment analyses, and Fisher’s exact test was used to obtain the most relevant experimental treatments for the biological pathways.

## RESULTS

### Changes in the intestinal flora of drones during sexual maturity

**Fig 1:**
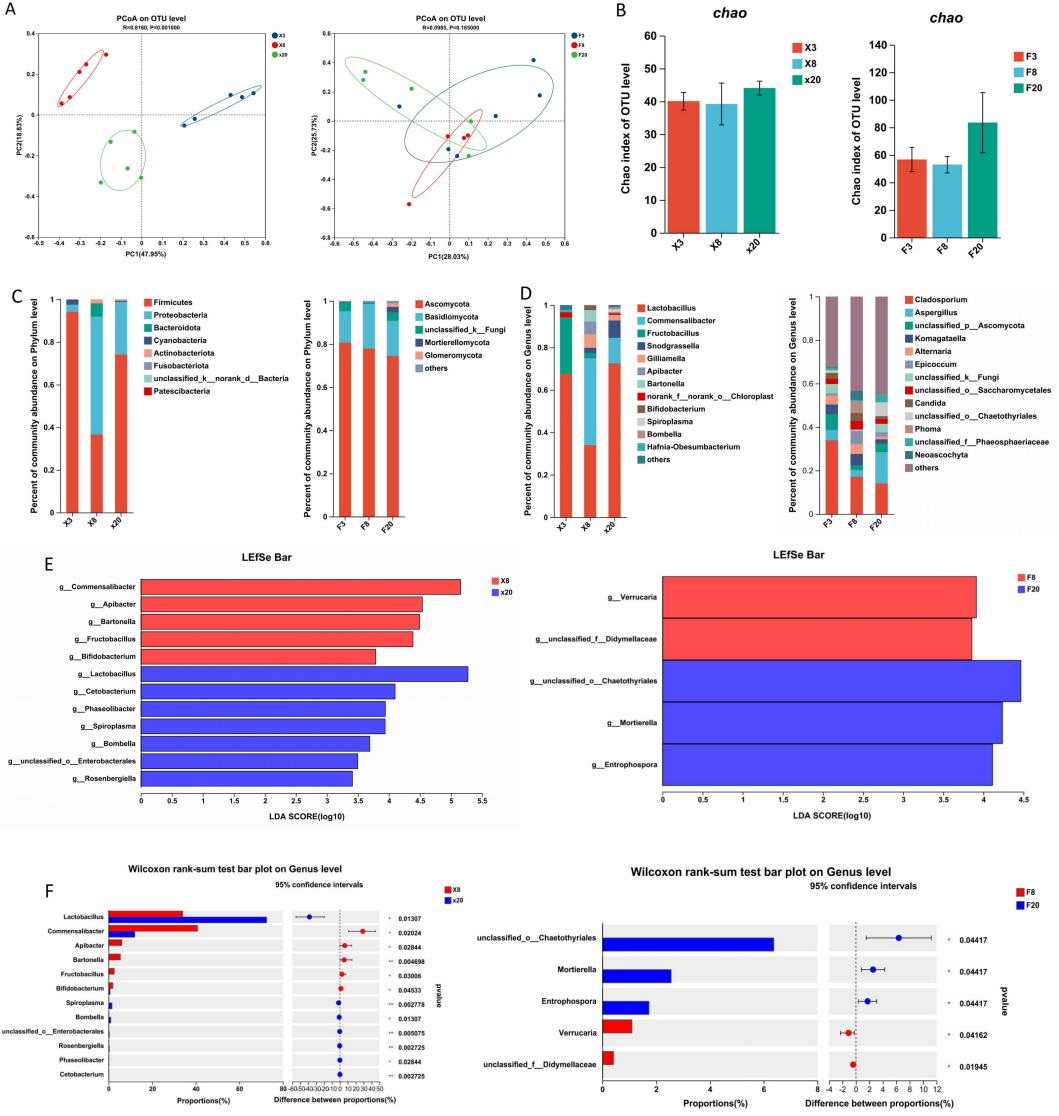
(**A**) PCoA of the gut microbiota of drones at OTU level, bacteria on the left, fungi on the right. (**B**) The Chao index of intestinal alpha diversity in drones, bacteria on the left, fungi on the right. *P < 0.05, **P < 0.01, and ***P < 0.0001. (**C**) Community distribution of intestinal bacteria at the phylum and genus levels in drones. (**D**) Community distribution of intestinal fungi of drones at phylum and genus levels. (**E**) LDA discriminant bar graphs summarizing microbial taxa with significant effects in the gut microbes of each group of drones, bacteria on the left, fungi on the right. *P < 0.05 and **P < 0.01. (**F**) Bar graph showing differences in mean relative abundance of the same species between groups, bacteria on the left, fungi on the right. *P ≤ 0.05, **P ≤ 0.01, and ***P ≤ 0.001. LDA, linear discriminant analysis; LEfSe, linear discriminant analysis effect size; PCoA, principal coordinate analysis.

We use principal coordinate analysis (PCoA) to explore the differences in the gut microbiota of drones during the sexual maturation process. The findings demonstrated that sexual maturation significantly altered the composition and distribution of gut microbiota in drones. A distinct trend of segregation was evident among gut bacteria in groups X3, X8, and X20, while a similar trend was observed among gut fungi in groups F3, F8, and F20, although the separation was less pronounced compared to bacteria (Fig. 1A). Thus, significant changes in bacterial and fungal populations were observed during the sexual maturation of drones.

Second, we used the Chao index to assess the difference in the abundance of gut microbiota before and after sexual maturity in drones. No significant difference in the Chao index was observed among the three groups using the Kruskal-Wallis rank sum test. Before sexual maturity, the X8 group exhibited a slight decrease in Chao index compared to the X3 group. In contrast, the X20 group demonstrated greater gut microbiota richness than both the X8 and X3 groups, with a similar pattern of fungal richness change to that of bacteria. The findings indicate that gut microbiota richness is elevated in sexually mature drones, with fungi showing higher abundance relative to bacteria (Fig. 1B).

At the level of phylum and genus, gut microorganisms differed significantly in drones before and after sexual maturity. For bacteria, at the phylum level, the predominant phyla in groups X3, X8, and X20 were Firmicutes (94.20%), Proteobacteria (55.26%), and Firmicutes (74.11%), respectively. At the genus level, *Lactobacillus* (67.46%), *Commensalibacter* (40.83%), and *Lactobacillus* (72.50%) were predominant in groups X3, X8, and X20, respectively (Fig. 1C). For fungi, at the phylum level, groups F3, F8, and F20 were dominated by Ascomycota with percentages of 80.71%, 77.93%, and 74.55%, respectively. At the genus level, the predominant fungi were *Cladosporium* (33.86%), *Cladosporium* (17.13%), and *Aspergillus* (14.28%) in groups F3, F8, and F20, respectively (Fig. 1D). From the above, it is clear that drones are dominated by Firmicutes and Ascomycota at the phylum level, and *Lactobacillus* and *Aspergillus* at the genus level, respectively, after sexual maturity.

Utilizing linear discriminant analysis effect size analysis, we determined differential intestinal flora between the two groups (Fig. 1E). The findings indicated higher levels of *Commensalibacter* and *Verrucaria* in X8 and F8, respectively, and elevated levels of *Lactobacillus* and unclassified *Chaetothyriales* in X20 and F20, respectively. Additionally, the diversity of gut flora was found to be greater in X20 compared to F20 than in X8 compared to F8. Consequently, the findings imply that the diversity of gut flora may be heightened in drones post-sexual maturation, and the core flora of drones may undergo changes as an adaptation to physiological alterations during sexual maturation in drones.

To further validate whether these differential gut flora existed between days 8 and 20, there was a significant difference among the groups. The Kruskal-Wallis *H* test was employed to create a bar plot for discrimination (Fig. 1F). The results revealed significant differences in *Lactobacillus*, *Chaetothyriales*, *Mortierella*, and *Entrophospora* between days 8 and 20 (*P* < 0.05). This suggests that these intestinal florae might play a role in the sexual maturation process of drones. Particularly, *Lactobacillus* and *Chaetothyriales* exhibited the most pronounced and statistically significant differences (*P* < 0.05) between the two groups. Consequently, they can serve as distinctive markers for the sexual maturation process of drones.

We utilized correlation network analysis to investigate the interaction between bacteria and fungi on days 8 and 20. Analyzing the top 50 nodes in terms of abundance (Fig. S1 and S2), the results revealed a strong positive correlation between *Apibacter* and *Snodgrassella* and the fungus *Cylindrobasidium* on day 8. *Bartonella* was also correlated with two fungi, *Ascomycota* and *Cladosporium*. On day 20, *Lactobacillus* did not exhibit any positive correlation with other strains. In contrast, *Chaetothyriales* showed positive correlations with *Mortierella*, *Entrophospora*, *Inocybe*, *Ascomycota*, *Ganodermataceae*, and *Geopora*, indicating associations with six species of fungi (Tables S1 and S2). These findings suggest that, following sexual maturation, *Lactobacillus* did not exhibit interactions with other strains. In contrast, *Chaetothyriales* displayed correlations with numerous fungi. However, the specific role of this interaction in the sexual maturation process of drones remains unclear.

### Changes in brain metabolites during sexual maturation in drones

**Fig 2:**
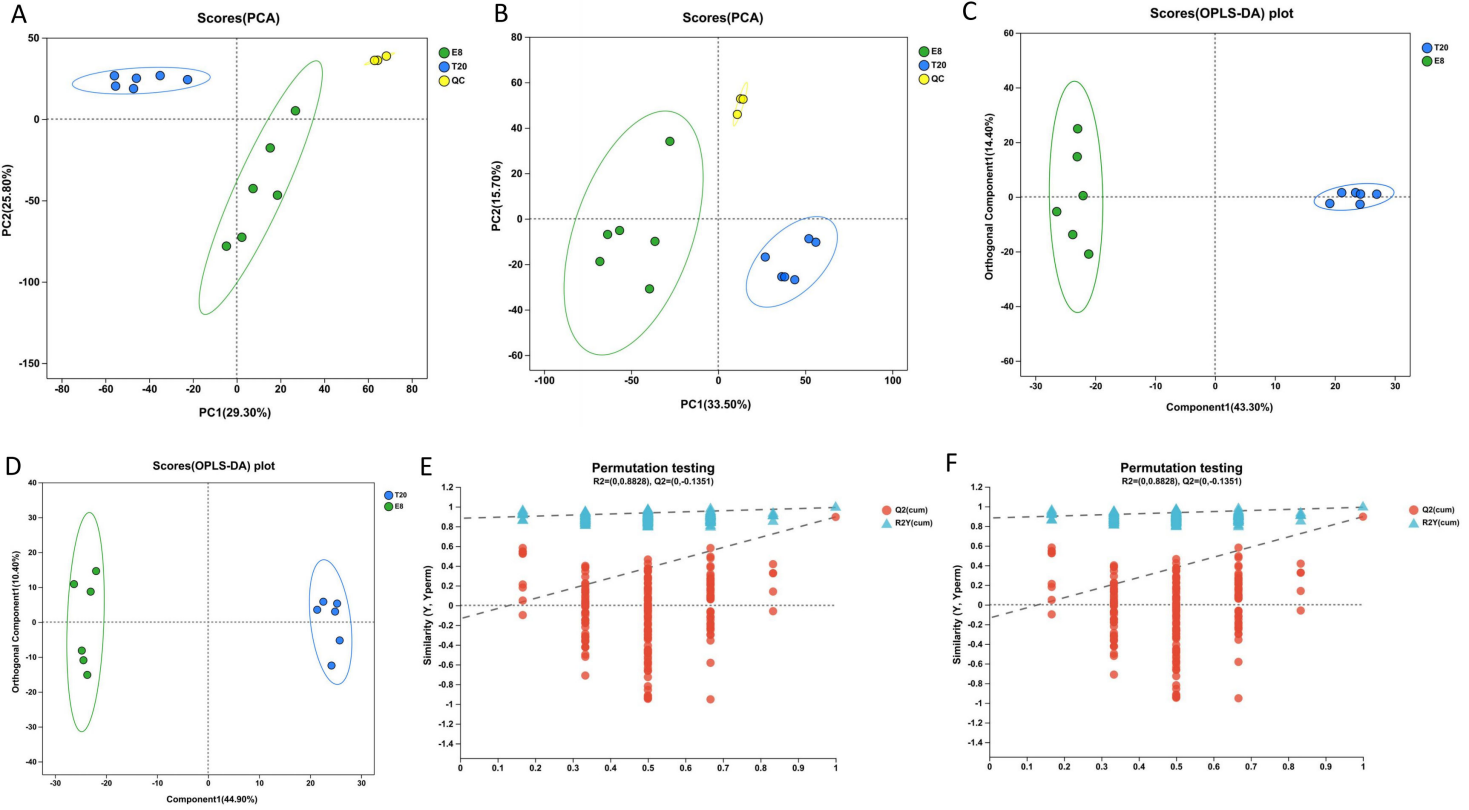
(A and B) The PCA score plots are shown in positive and negative ion modes, separately. (**C and D**) The OPLS-DA score plots are shown in positive and negative ion modes, separately. (**E and F**) The OPLS-DA substitution test plots are shown in positive and negative ion modes, respectively. OPLS-DA, orthogonal partial least squares discriminant analysis; PCA, principal component analysis.

UHPLC-MS was employed to analyze brain metabolites from the N8 and T20 groups. Post-analysis, a total of 1,843 metabolites encompassing lipids, carbohydrates, antibiotics, peptides, vitamins, cofactors, organic acids, hormones, and transmitters were detected in both sample sets (Table S3). Utilizing this metabolite information, PCA was employed to initially assess the overall metabolic variances between N8 and T20, as well as the variability within the sample groups. The PCA score plots revealed a distinct separation between N8 and T20 in both anionic and cationic modes, along with minor variability within the sample groups. This indicates variations in the composition of brain metabolites in drones during sexual maturation (Fig. 2A and B).

To enhance the characterization of these metabolites, OPLS-DA was conducted. The OPLS-DA score plots demonstrated a distinct separation between N8 and T20 in both anionic and cationic modes (Fig. 2C and D). Moreover, to prevent overfitting, the OPLS-DA results underwent evaluation through the OPLS-DA substitution test, revealing an *R*^2^ of 0.8828 in the cationic mode and 0.8998 in the anionic mode (Fig. 2E and F). These findings suggest that the OPLS-DA model exhibits relative reliability in both cationic and anionic modes, and it can be used for further analysis of differential metabolites.

We selected these differential metabolites based on a combination of statistical significance thresholds for predictor variable effect (VIP) values derived from the OPLS-DA model and *P* values derived from a two-tailed Student’s *t* test for normalized peak area. In addition, metabolites with VIP values greater than 1.0 and *P* values less than 0.05 were considered statistically significant.

**Fig 3:**
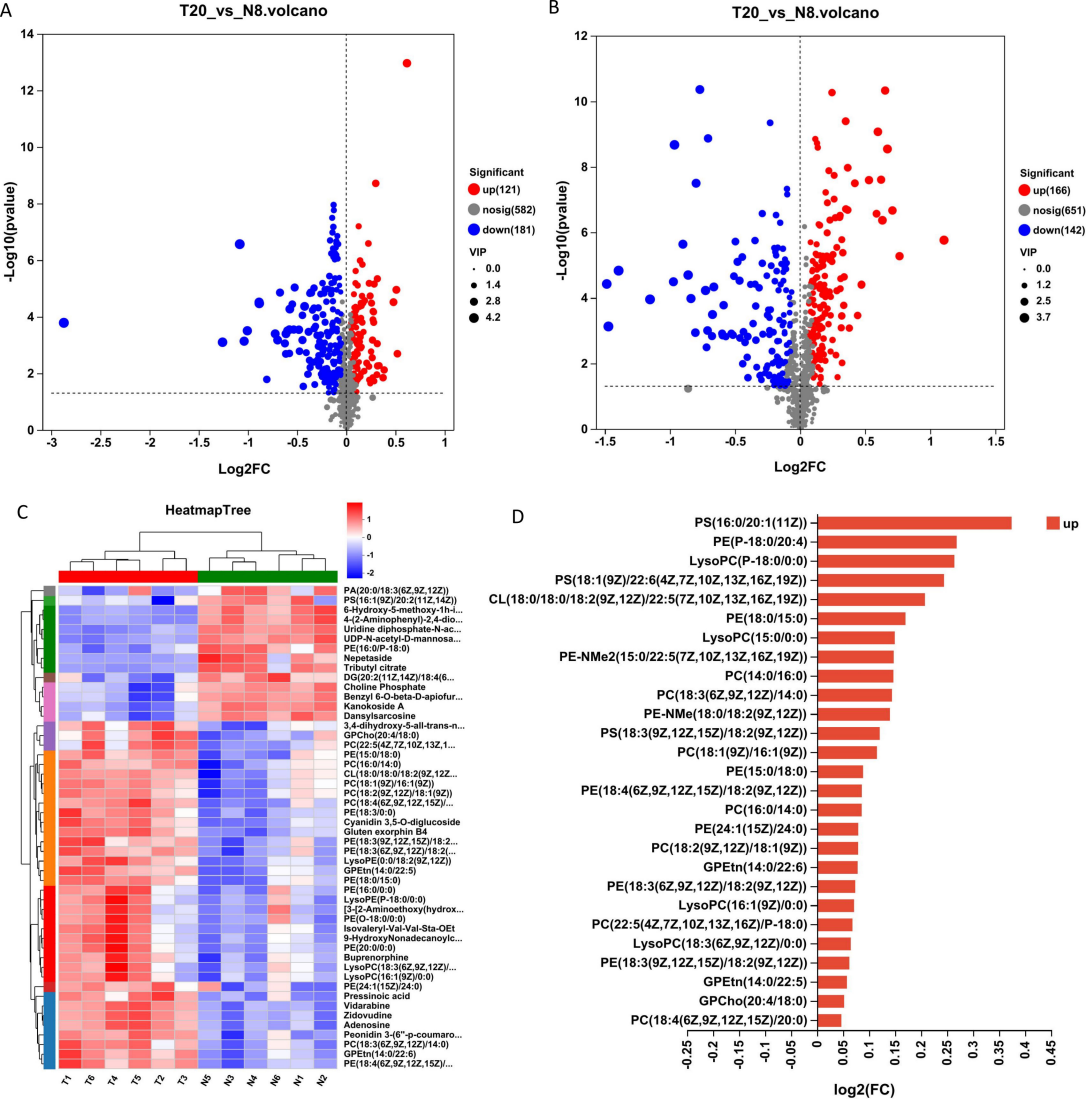
(A and B) The volcano plots are shown in positive and negative ion modes, separately. (**C**) The clustering analysis of each sample with the top 50 abundant metabolites, with red indicating positive correlation and blue indicating negative correlation. (**D**) Differential multiplicity bar graphs of upregulated substances in the glycerophospholipid metabolic pathway.

Based on these differential metabolites, volcano plots were constructed to specifically display the metabolites within the two groups. In the cation mode volcano plot, a total of 1,707 differential ion peaks were detected, with only 302 metabolites identified (Fig. 3A). Similarly, in the anion mode volcano plot, 1,707 differential ion peaks were detected, resulting in the identification of only 308 metabolites (Fig. 3B). The volcano plot revealed higher values of fold change in metabolite expression differences between the two groups in the cation mode compared to the anion mode. However, no significant difference was observed in the statistical test values for metabolite expression changes between the two groups. A total of 611 named metabolites were detected, with 287 being upregulated and 323 downregulated (Table S4).

To better understand these differential metabolite changes in the brain, we selected the 50 most abundant metabolites to construct a clustered heat map (Fig. 3C). The results showed that the expression of 36 metabolites was increased in the T20 group and only 14 metabolites were downregulated, which was contrary to the previous results for differential metabolites. In addition, 18 of the 36 metabolites that increased belonged to the glycerophospholipid metabolic pathway (Table S5), suggesting that during sexual maturation of drones, although the expression of differential metabolites was downregulated more in the brain, the upregulation of expression was dominant in metabolites of higher abundance, and the expression of glycerophospholipid pathway-related substances was increased among them. Upregulated substances in the brain are closely associated with reproductive behavior in sexually mature drones (18), with significant enrichment in the glycerophospholipid metabolic pathway. This indicates that it plays a significant role in the reproductive behavior of sexually mature drones. We used the upregulated substances in the glycerophospholipid metabolic pathway to draw a multiplicity-of-difference bar graph, as shown in Fig. 3D, and the results showed that PS (16:0/20:1 [11Z]), PE (P-18:0/20:4), and LysoPC (P-18:0/0:0) were highly variable, and therefore are expected to be markers related to the reproductive behavior of sexually mature drones.

**Fig 4:**
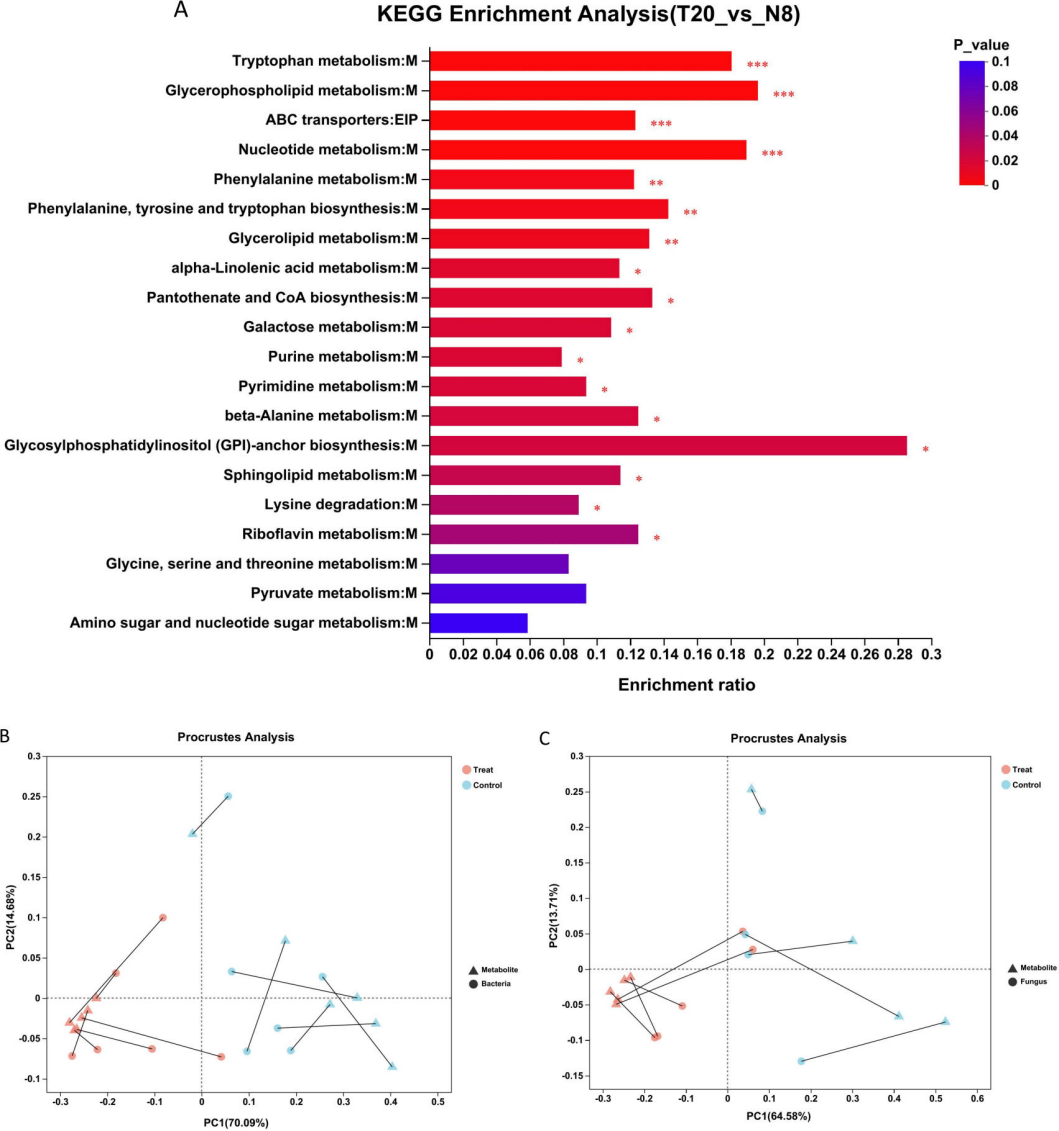
(**A**) KEGG enrichment analysis plot. The column color gradient indicates the significance of enrichment, where the darker the default color, the more significantly enriched the KEGG term is, where *P* value or FDR < 0.001 is marked as ***, *P* value or FDR < 0.01 is marked as **, and *P* value or FDR < 0.05 is marked as *. (B and C) Procrustes analysis of gut bacteria and fungi with metabolites.

To gain deeper insights into the alterations occurring in the brain of drones following sexual maturation, pathway enrichment analysis was conducted on differential metabolites, resulting in the enrichment of 62 pathways (Table S6). These pathways encompass glycerophospholipid metabolism, tryptophan metabolism, nucleotide metabolism, ABC transporter proteins, phenylalanine metabolism, and pyrimidine metabolism (Fig. 4A). Specifically, glycerophospholipid metabolism, tryptophan metabolism, nucleotide metabolism, and ABC transporter proteins, along with phenylalanine metabolism that incorporating phenylalanine, tyrosine, and tryptophan biosynthesis (*P* < 0.001), as well as glycerophospholipid metabolism, purine metabolism, and pyrimidine metabolism (*P* < 0.01), showed significant impacts. Analysis of the metabolites associated with glycerophospholipid metabolism indicated an upregulation in the expression of 27 substances and a downregulation in 12 substances, which included choline phosphate and glycerol 4-phosphate (Table S7).

### Correlation analysis between gut microbes and brain metabolites

To investigate whether there is a significant correlation between gut microbes and brain metabolites as a whole, we discussed the overall correlation between the two using Procrustes analysis. The results showed that the *P* value between gut bacteria and brain metabolites was less than 0.01, with an M2 value of 0.482 (Fig. 4B; Table S8), suggesting that the trend between gut bacteria and brain metabolites was highly significant and consistent across the groups and that the degree of correlation between these two data sets was high. The *P* value between gut fungi and brain metabolites was greater than 0.05 with an M2 value of 0.706 (Fig. 4C; Table S9), indicating that the trend between gut fungi and brain metabolites was not significant across groups and that the degree of association between these two data sets was low. Thus, it can be seen that there is a significant correlation between gut bacteria and brain metabolites with a greater effect than fungi.

**Fig 5:**
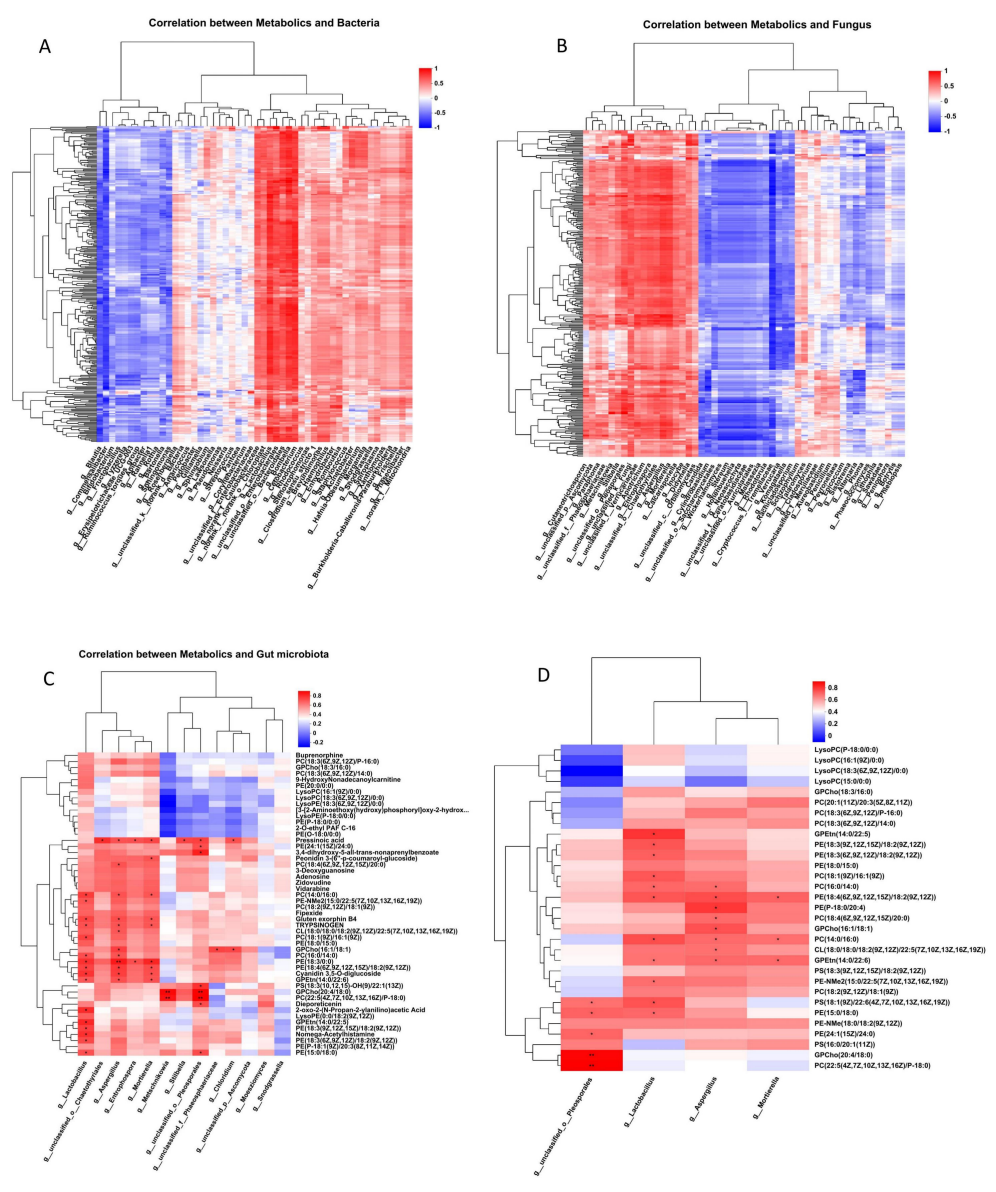
(A and B) Correlation analysis of 287 upregulated substances with top 50 gut bacteria and fungi in abundance using the Pearson correlation algorithm. (**C**) Correlation analysis of 13 drones gut flora with the top 50 metabolites in abundance; the darker the color, the greater the correlation coefficient. (**D**) Correlation analysis was performed between four differential gut flora and upregulated substances in the glycerophospholipid pathway; the darker the color, the greater the correlation coefficient.

Substances that increase in the brain of drones during sexual maturation have important implications for reproductive behaviors such as flight and learning memory in dron*es* (18), so we used the Pearson correlation algorithm to calculate the correlation between the top 50 intestinal flora with the highest abundance and 287 upregulated metabolites (Table S10) and created a correlation analysis heat map (Fig. 5A and B). The plot indicated strong correlations between *Fructobacillus*, *Tyzzerella*, *Blautia*, *Erysipelotrichaceae_UCG*, *Ruminococcus_torques_group*, *Commensalibacter*, *Chloroplast*, *Lactobacillus*, *Bombella*, *Cetobacterium*, *unclassified_o__Bacteroidales,* and 287 metabolites. Meanwhile, other gut bacteria displayed partial involvement in regulating metabolic activity in the brains of drones. Regarding fungi, the findings revealed robust correlations between *Cryptococcus_f__Tremellaceae*, *Wickerhamomyces*, *Stereum*, *Hohenbuehelia*, *Neoascochyta*, *Ceratobasidiaceae*, *unclassified_o__Auriculariales*, *Entrophospora*, *Apiotrichum*, *Aspergillus*, *unclassified_o__Chaetothyriales*, *Metschnikowia*, *unclassified_o__Pleosporales*, *Stilbella*, and 287 metabolites. The findings imply that the gut microbiota of drones may impact the metabolic activity of the brain. Nonetheless, their influence seems somewhat constrained, as the brain itself seems to exert a more predominant regulatory function.

To further explore the correlation between gut flora and brain metabolites, we selected *Lactobacillus*, *Snodgrassella*, *Chaetothyriales*, *Pleosporales*, *Mortierella*, and *Entrophospora* from the drone gut microbiota, as well as *Metschnikowia*, *Moesziomyces*, *Chloridium*, *Stilbella*, *Aspergillus*, *Ascomycota*, and *Phaeosphaeriaceae*. The Pearson correlation algorithm was utilized to assess the relationship between the 13 gut microbiota and the top 50 most abundant substances among 287 substances, followed by the construction of a correlation heat map (Fig. 5C). The results indicated significant correlations between *Lactobacillus*, *Aspergillus*, *Mortierella*, *Chaetothyriales*, *Pleosporales*, and *Stilbella* and substances in the glycerophospholipid metabolic pathway; among them, *Lactobacillus*, *Aspergillus*, *Mortierella*, and *Pleosporales* have the strongest correlation, implying their potential role in influencing these compounds during sexual maturation.

From the above results, it can be seen that these gut microbiota are closely related to substances in the glycerophospholipid metabolic pathway. In particular, *Lactobacillus*, *Aspergillus*, *Mortierella*, and *Pleosporales*. Therefore, we explored the correlation between the above four gut microbiota and substances in the glycerophospholipid metabolic pathway (Fig. 5D). The results showed that *Lactobacillus*, *Aspergillus*, *Mortierella*, and *Pleosporales* were significantly correlated with substances such as PC and PE. Among them, *Lactobacillus* correlated with the most substances. Therefore, it indicates that *Lactobacillus* may play a major role.

From the above results, it is evident that a substantial correlation exists between the gut flora and brain metabolites in drones. Bacteria and fungi, notably *Lactobacillus*, *Aspergillus*, *Mortierella*, and *Pleosporales*, seem to play a role in the sexual maturation process of drones primarily through their impact on glycerophospholipid metabolism.

## DISCUSSION

In this study, we found that the highest relative abundance of intestinal flora of drones after sexual maturity was *Lactobacillus* and *Aspergillus*, with 72.51% and 14.28%, respectively. Moreover, *Snodgrassella* exhibited an increase from 2.42% to 8.15%. *Lactobacillus* plays significant probiotic roles, including enhancing immunity (19), assisting bees in resisting pests and diseases (20), improving sperm quality (21), and modulating learning and memory in bees through the regulation of tryptophan metabolism (22). Additionally, *Lactobacillus* influences glycerophospholipid metabolism in the hemolymph of bumblebees, thereby enhancing long-term memory (23). Furthermore, an examination of the intestinal flora of sexually mature male rhesus monkeys indicated that *Lactobacillus* significantly increased after sexual maturity (24). Consequently, *Lactobacillus* may play a critical role in the sexual maturation process of drones, particularly in enhancing learning and memory among sexually mature males. *Snodgrassella*, a core component of the gut flora in drones (25), has been shown to enhance bumblebee resistance to *Crithidia bombi* (26).

In addition, *S. alvi* plays an important role in digesting carbohydrates such as honey and pollen. Thus, an increase in *S. alvi* may provide sexually mature drones with enhanced resistance to pathogens and a greater energy supply. *Aspergillus*, a microorganism prevalent in western honeybee colonies (*Apis mellifera*), is frequently found in bee bread and is believed to play a significant role in the processing, preservation, and digestion of honeybee food (27). Nevertheless, the role of sexually mature drones remains unclear and needs further investigation. Through Lefse analysis, we found that *Commensalibacter* and *Verrucaria* were enriched in day 8 drones, while *Lactobacillus* and *Chaetothyriales* were enriched in day 20 drones. These strains, enriched on days 8 and 20, serve as key biomarkers for sexual maturity in drones. To further identify strains associated with sexual maturation, we evaluated the significance of differences between groups for these strains. The results reported that the mean relative abundance of *Lactobacillus* and *Chaetothyriales* differed significantly between days 8 and 20. Therefore, these two gut microorganisms can be used as microbial markers for sexual maturation in drones.

Additionally, correlation network analysis revealed that *Lactobacillus* did not exhibit a positive correlation with the remaining strains. In contrast, *Chaetothyriales* showed positive correlations with *Mortierella*, *Entrophospora*, *Inocybe*, *unclassified_p__Ascomycota*, *unclassified_f__Ganodermataceae*, and *Geopora*. This suggests that *Chaetothyriales* may play a part in the sexual maturation process of drones through interactions with these other fungi.

Glycerophospholipids are cardinal components of nerve membranes and play a critical role in their function and structure (28). Research has shown that low levels of glycerophospholipids are associated with memory deficits (22, 23). While supplementation with these lipids enhances cognitive function in humans, rats, and mice (29). Furthermore, administering glycerophospholipids to bumblebees has been demonstrated to enhance their long-term memory (4). Therefore, glycerophospholipids exert a meaningful influence on memory function. Our results indicate that the glycerophospholipid metabolic pathway is significantly enriched in the brains of drones during sexual maturation, predominantly comprising phosphatidylcholine (lecithin, PC) and phosphatidylethanolamine (ceruloplasmin, PE). The levels of these substances have been positively correlated with memory performance in honeybees (4, 24). Additional studies have demonstrated that phosphatidylcholine increases cerebral acetylcholine concentrations and enhances memory in mice with dementia (30). Consequently, elevated levels of substances such as PC and PE may impact memory in drones during sexual maturation; however, further experimental verification is required.

**Fig 6:**
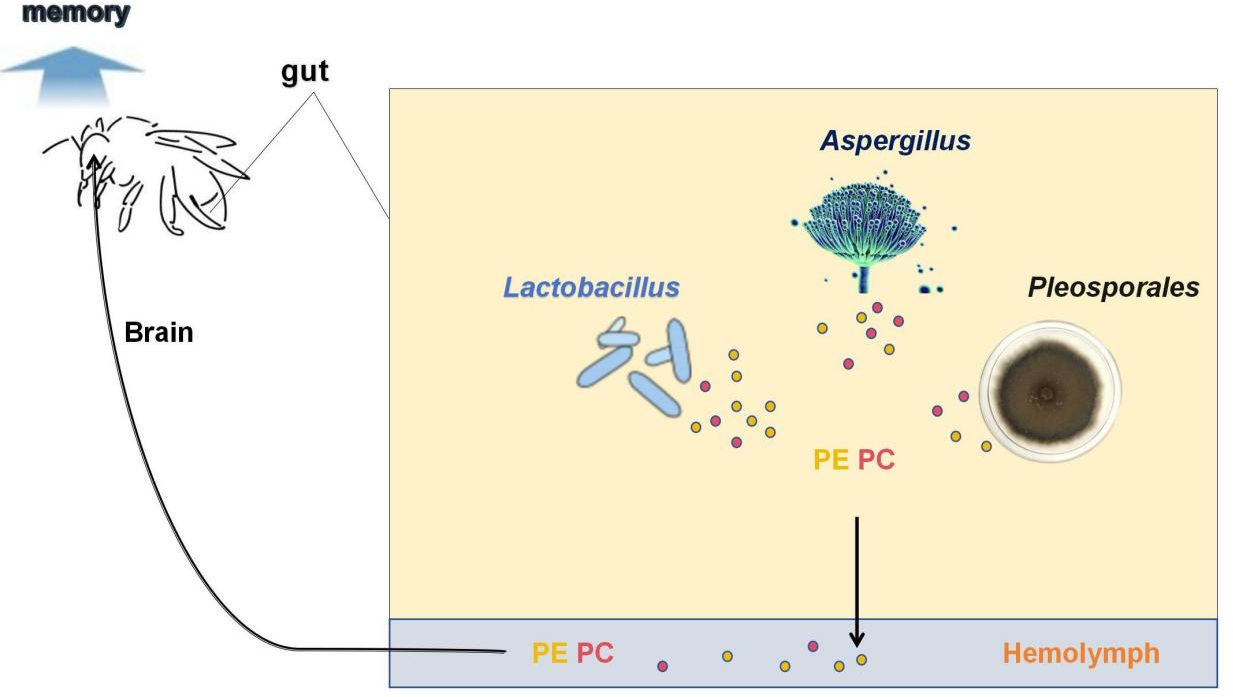
Hypothesized pathways between gut microbiota, glycerophospholipids, and brain memory. Based on our analysis of gut flora and brain metabolomics, along with related studies, we propose the following hypothesis. A significant increase in the abundance of *Lactobacillus*, *Aspergillus*, and *Pleosporales* following sexual maturation leads to enhanced production of glycerophospholipids. These substances are translocated from the hindgut into the hemolymph through specific transport pathways. Subsequently, glycerophospholipids reach the brain and enhance memory function in drones.

Intestinal flora can influence the host’s memory; it is derived from their ability to produce and secrete associated neurotransmitters (31). *Lactobacillus* has been shown to impact host memory primarily by altering pathways associated with glucose metabolism, amino acid metabolism, and glycerophospholipid metabolism, and producing indole derivatives and memory-enhancing metabolites, such as PC and PE (4, 31). Through correlation analysis, it was determined that *Lactobacillus*, *Aspergillus*, and *Pleosporales* exhibited strong correlations with PC and PE in the glycerophospholipid metabolic pathways within the brains of drones. Consequently, *Lactobacillus*, *Aspergillus*, and *Pleosporales* may enhance the memory of drones by producing greater quantities of PC and PE during sexual maturation. Depending on Li et al., *Lactobacillus*, particularly *Lactobacillus* Firm-5, can increase glycerophospholipid content in bumblebee hindgut and hemolymph. This increase may be attributed to the presence of PTS genes in *Lactobacillus* Firm-5, which enhance the uptake of fructose and cellobiose. These sugars are converted to d-Fructose-1,6P_2_, which is further metabolized to glyceraldehyde-3P and glycerone-P, ultimately leading to the production of more glycerophospholipids. The glycerophospholipids are then translocated to the hindgut and quickly secreted into the hemolymph, possibly headed by an increase in the metabotropic glutamate receptor 2. They are transported to the brain via the honeybee’s open circulatory system, along with other metabolites, to enhance the structure and function of neural and synaptic membranes, ultimately improving memory. Therefore, based on the strong correlations observed between the aforementioned three gut microorganisms and PC and PE, in conjunction with the study by Li et al. (4) and related research, we propose a potential mechanism of action wherein *Lactobacillus*, *Aspergillus*, and *Pleosporales* proliferate significantly during the sexual maturation of drones, subsequently producing increased levels of PC and PE through specific metabolic pathways. These substances are secreted into the hemolymph via specific transport pathways in the intestine and subsequently transported to the brain, ultimately influencing the memory of drones (Fig. 6).

### Conclusion

In this study, we analyzed gut microbes and brain metabolites before and after sexual maturation in drones. We found that gut microbial diversity was elevated and core gut microbes changed after sexual maturation in drones. Specifically, the relative abundance of *Lactobacillus* rose from 34.02% to 72.50%. This rise may have enhanced learning and memory as well as flight ability in sexually mature drones, among other things. From the perspective of brain metabolites, drones exhibited increased metabolic activity after sexual maturity, especially glycerophospholipid metabolism. Through comprehensive analysis, we found that *Lactobacillus* in the gut microbes of drones may regulate the brain metabolism of drones through glycerophospholipid metabolism.

## Data Availability

The 16S rRNA and ITS raw data of the drone have been deposited in the NCBI database under BioProjects PRJNA949168 and PRJNA1187012, respectively. Metabolome raw data have been uploaded to MetaboLights under MTBLS11708.

## SUPPLEMENTAL MATERIAL

Supplemental figures spectrum.02536-24-s0001.docx Fig. S1 and S2.

Supplemental tables spectrum.02536-24-s0002.xlsx Tables S1 to S10.

ASM does not own the copyrights to Supplemental Material that may be linked to, or accessed through, an article. The authors have granted ASM a non-exclusive, world-wide license to publish the Supplemental Material files. Please contact the corresponding author directly for reuse.
