# Supplementary material for: Gut microbiota regulates the brain metabolism of sexually mature drones
Source: Microbiol Spectr. 2025 Jun 5;13(7):e02536-24. doi: 10.1128/spectrum.02536-24 (PMC12210970; doi:10.1128/spectrum.02536-24)
Supplement: Supplemental figures — Fig. S1 and S2. [file spectrum.02536-24-s0001.docx]

**
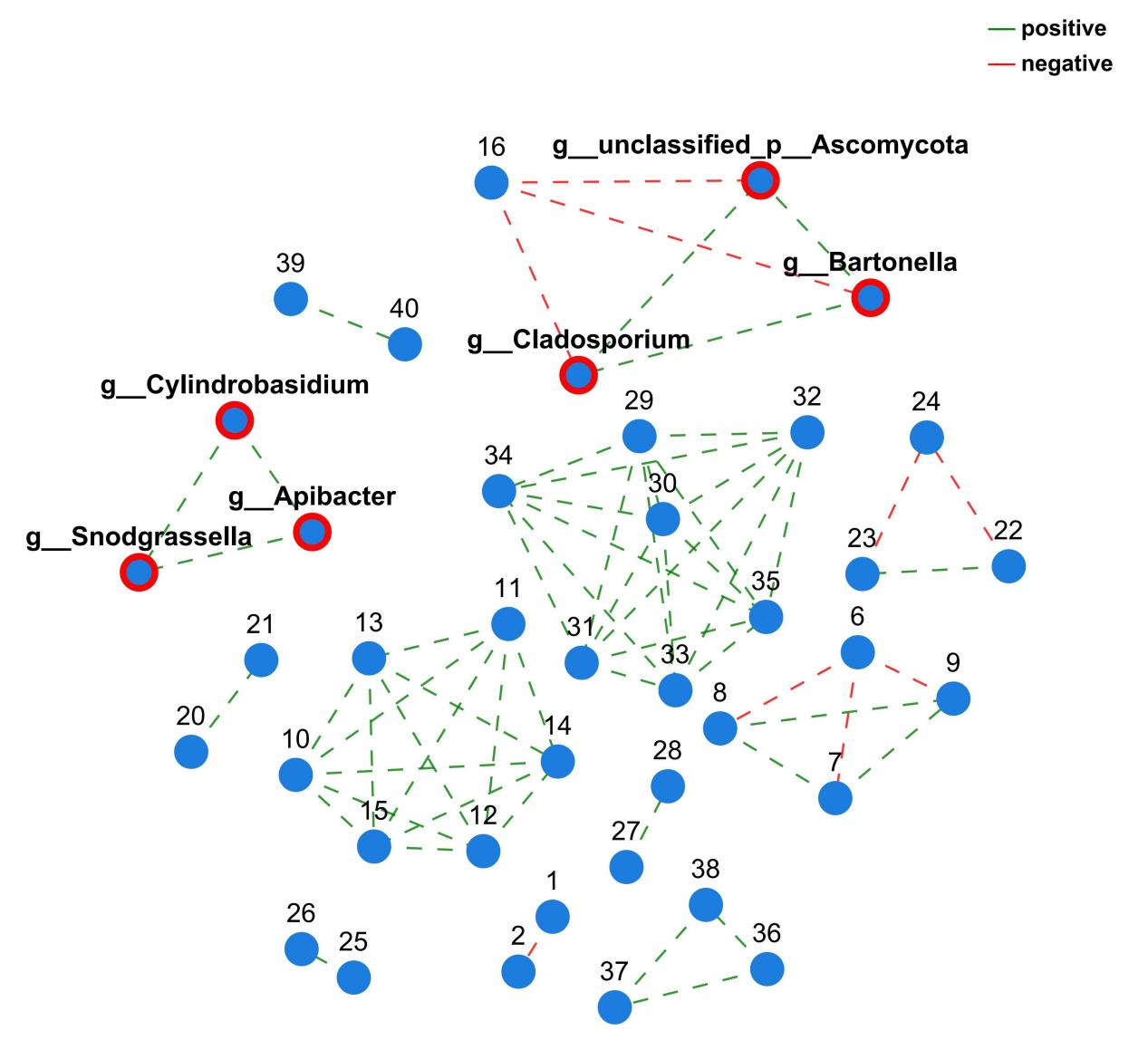
**

**Supplementary Fig. S1.** Correlation networks constructed with gut bacterial species and fungi show different abundances at d8. Connecting lines between nodes indicate Spearman negative (light red) or positive (light green) correlations.

**
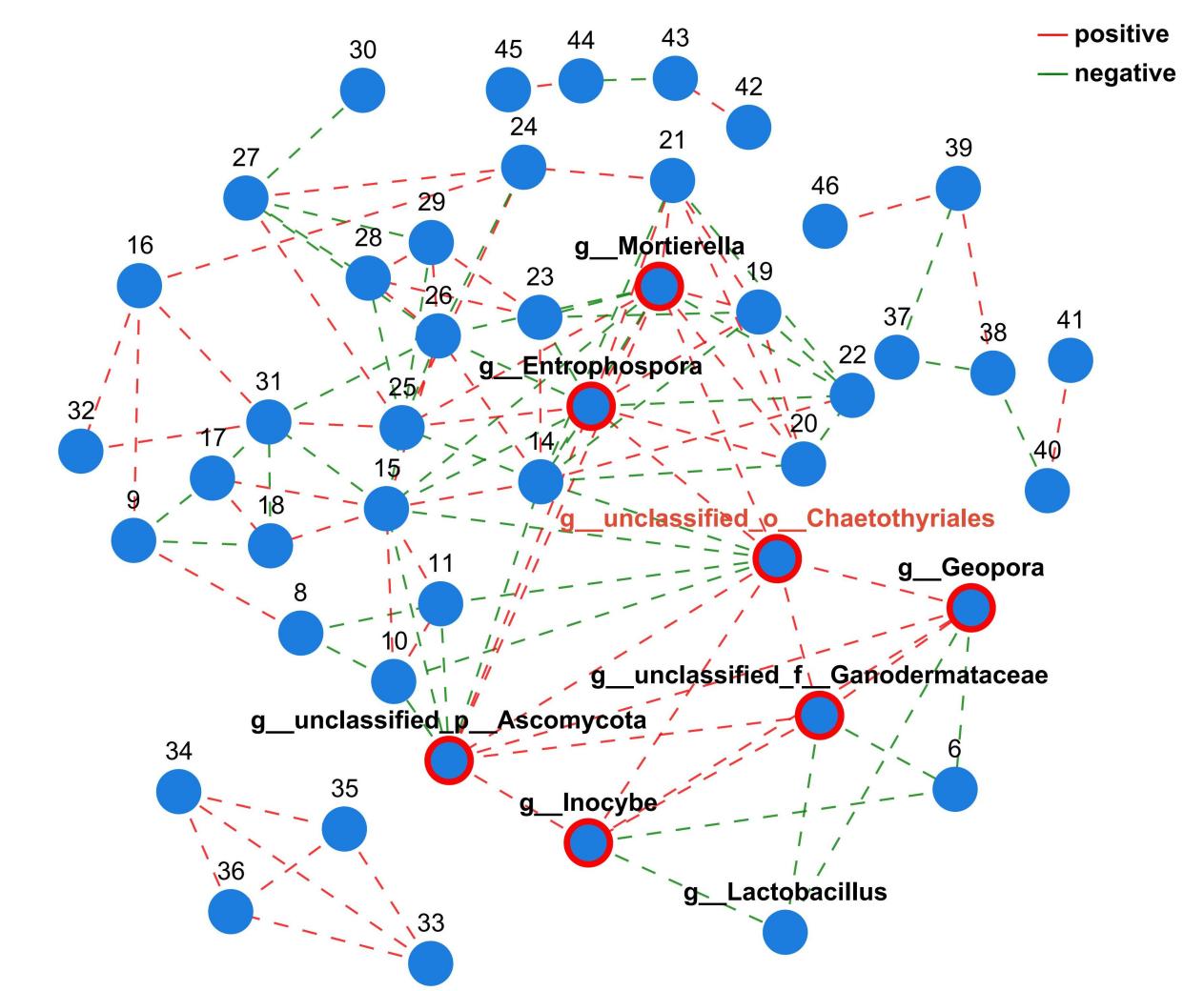
**

**Supplementary Fig. S2.** Correlation networks constructed with gut bacterial species and fungi show different abundances at d20. Connecting lines between nodes indicate Spearman negative (light green) or positive (light red) correlations.
